# AI assistance in aesthetic medicine–A consensus on objective medical standards

**DOI:** 10.1111/jocd.16481

**Published:** 2024-08-01

**Authors:** Konstantin Frank, Doris Day, Julius Few, Chhabra Chiranjiv, Michael Gold, Sonja Sattler, Martina Kerscher, Leonard Knoedler, Alexandre Filippo, Berthold Rzany, Sebastian Cotofana, Sabrina Fabi, Klaus Fritz, Peter Peng, Rungsima Wanitphakdeedecha, Rainer Pooth, Patrick Huang

**Affiliations:** ^1^ Department of Plastic, Hand and Reconstructive Surgery University Hospital Regensburg Regensburg Germany; ^2^ New York University Langone Health Medical Centers New York New York USA; ^3^ University of Chicago Pritzker School of Medicine Chicago Illinois USA; ^4^ Skin Alive Clinics New Delhi India; ^5^ Gold Skin Care Center, Tennessee Clinical Research Center Nashville Tennessee USA; ^6^ Rosenpark Klinik Darmstadt Germany; ^7^ Department of Chemistry, Division of Cosmetic Sciences University of Hamburg Hamburg Germany; ^8^ Department of Plastic Hand and Reconstructive Surgery, University Hospital Regensburg Regensburg Germany; ^9^ Instituto de Dermatologia Prof. Rubem David Azulay Rio de Janeiro Brazil; ^10^ Medizin am Hauptbahnhof, Wien, Austria and Hautarzt Friedenau Berlin Germany; ^11^ Department of Dermatology Erasmus Medical Centre Rotterdam The Netherlands; ^12^ University of California San Diego San Diego California USA; ^13^ Dermatology and Laser Center Landau in der Pfalz Germany; ^14^ P‐Skin Professional Clinic Taipei Taiwan; ^15^ Department of Dermatology, Faculty of Medicine Siriraj Hospital Mahidol University Bangkok Thailand; ^16^ Clinical Research and Development, ICA Aesthetic Navigation GmbH Frankfurt Germany; ^17^ Huang PH Dermatology and Aesthetics Taipei Taiwan

**Keywords:** aesthetic medicine, artificial intelligence, consensus meeting, medical technology, objective standards

## Abstract

**Background:**

Aesthetic medicine has traditionally relied on clinical scales for the objective assessment of baseline appearance and treatment outcomes. However, the scales focus on limited aesthetic areas mostly and subjective interpretation inherent in these scales can lead to variability, which undermines standardization efforts.

**Objective:**

The consensus meeting aimed to establish guidelines for AI application in aesthetic medicine.

**Materials and Methods:**

In February 2024, the AI Consensus Group, comprising international experts in various specialties, convened to deliberate on AI in aesthetic medicine. The methodology included a pre‐consensus survey and an iterative consensus process during the meeting.

**Results:**

AI's implementation in Aesthetic Medicine has achieved full consensus for enhancing patient assessment and consultation, ensuring standardized care. AI's role in preventing overcorrection is recognized, alongside the need for validated objective facial assessments. Emphasis is placed on comprehensive facial aesthetic evaluations using indices such as the Facial Aesthetic Index (FAI), Facial Youth Index (FYI), and Skin Quality Index (SQI). These evaluations are to be gender‐specific and exclude makeup‐covered skin at baseline. Age and gender, as well as patients' ancestral roots, are to be considered integral to the AI assessment process, underlining the move towards personalized, precise treatments.

**Conclusion:**

The consensus meeting established that AI will significantly improve aesthetic medicine by standardizing patient assessments and consultations, with a strong endorsement for preventing overcorrection and advocating for validated, objective facial assessments. Utilizing indices such as the FAI, FYI, and SQI allows for gender‐specific, age adjusted evaluations and insists on a makeup‐free baseline for accuracy.

## 1 INTRODUCTION

In aesthetic medicine, the pursuit of objectivity in assessing baseline appearance and treatment outcomes has often been confined to the use of validated scales.^1^, ^2^, ^3^, ^4^ These scales, while being the closest tool to an objective metric currently available, are usually focusing on a specific anatomical area and inherently limited by their reliance on human evaluation. The efficacy of such scales is predicated on the subjective interpretation of the practitioner or patient using them, which introduces a layer of variability that is at odds with the goal of standardization. Each user brings their own set of biases and perceptions, which can influence the outcome of the scale's application, leading to inconsistencies in assessment and for example, treatment efficacy. The inherent variability in human perception makes it challenging to reach a universally accepted standard for beauty and treatment success.^1^, ^5^, ^6^ The need for an unbiased, automated system that can provide consistent and repeatable evaluations is evident, and it is here that AI's potential for impact is most keenly felt. By employing AI, we can overcome the limitations of human subjectivity and furthermore the limitation of considering isolated aesthetic and move towards more reliable and objective standards that take a holistic assessment of the human face into consideration.^7^

The integration of Artificial Intelligence (AI) into healthcare has marked a revolutionary pivot in the delivery of personalized, efficient, and cutting‐edge medical services. Fields such as radiology, pathology, dermatology, and now, aesthetic medicine, are on the way to experiencing significant enhancements through AI applications. This technology has become instrumental in diagnostics, patient monitoring, and predictive analytics, offering a fresh perspective on treatment strategies across various medical disciplines.^8^, ^9^, ^10^, ^11^

The promise of AI in aesthetic medicine extends beyond mere measurement; it has the potential to tailor treatments to individual preferences and objectively assess outcomes. Nonetheless, the use of AI in this field is not without its potential pitfalls and problems. Ethical considerations, such as privacy, consent, and bias, as well as practical issues concerning standardization and clinical integration, necessitate careful deliberation. To decide on the recommendations for the use of AI in aesthetic medicine and potential future AI directions we held a global consensus involving key opinion leaders and experts in the field of aesthetic medicine. The outcomes and discussions of this consensus meeting are presented in this paper.

## 2 CONSENSUS OBJECTIVES AND METHODOLOGY

In December 2023, an assembly of international experts across multiple specialties—plastic surgery and dermatology—formed an AI Consensus Group. The objective of this consensus was to create guidelines and recommendations for the development, use and application of AI within the field. Furthermore, the challenge of integrating and identifying ancestral roots into AI assessment was discusses, including a brief description of key characteristics of distinct ethnic roots. Prior to the consensus meeting an online survey was sent out to the participants in order to have a status‐quo on the use and perception of AI in aesthetic medicine which served as a base for the initial consensus statements (Table 1: Content of the pre‐meeting questionnaire). The consensus meeting which took place in February 2024 followed a structures approach involved not just passive agreement but active affirmation, with participants endorsing statements through verbal assent and a show of hands. If consensus could not be reached, the discourse continued with adjustments to the statement until a majority agreement was secured. This iterative process ensured that the guidelines developed were both robust and representative of the collective expertise present.

**TABLE 1:** Summary of pre–consensus questionnaire.

Consensus topic	Findings/statements	Count (*n*)	Notes
Facial examination strategies	Live examinations, photographs, personal experience, mathematical measurements, scales, AI, patient wishes, and economic abilities	Varied	Emphasis on a combination of strategies, including AI
AI assistance in aesthetic medicine	To prevent overcorrection, identify treatment options, achieve faster results, monitor treatments, work more effectively, and level up to other specialties	High importance	AI viewed as a tool to enhance efficiency and outcomes
Objectifying facial examination	Importance of introducing tools to objectify facial examination	Average importance 8.2 ± 1.1 (from 1 to 10)	High average importance rating
Clinical steps improvement	Patient examination, procedure selection, consultation, guidance, and follow‐up	Highest counts in patient examination and procedure selection	AI seen as particularly useful in early stages of patient interaction
Skin quality assessment	Differentiation between male and female skin quality, exclusion of makeup‐covered skin for AI examination	Majority agreement	Gender‐specific assessments and clean skin baseline needed
Holistic facial indices	Introduction of FAI, FYI, SQI to improve patient evaluation	High agreement	Novel indices supported for comprehensive assessments
Root‐specific assessment and treatment	Importance of integrating root‐specific features into evaluation and treatment	Majority agree it's important	Recognition of ethnic‐specific demands in treatment
Root‐guided AI algorithm refinement	Adapting AI algorithms to patient categories for holistic evaluation	High importance	Call for personalized AI algorithms based on patient roots.
Research and future directions	Areas that would benefit from AI: objective patient evaluation, personalized treatment plans	High anticipation	Acknowledgement of AI's potential in future research
Ethical considerations	Role of AI in decision‐making, how to proceed when AI recommendations differ from human judgment	Mixed responses	Discussion on AI as an assistive versus autonomous tool
AI application usability	User‐friendliness of CAARISMA application interface for providers and patient response	Neutral to very user‐friendly	Positive reception overall, with some neutrality
Expanding scope	Using CAARISMA for broader applications in aesthetic and reconstructive medicine	Suggested	Calls for broader applications and access to AI tools

## 3 RESULTS OF PRE–CONSENSUS SURVEY

Survey findings indicate minimal AI use for patient analysis in aesthetic medicine, with most practitioners prioritizing patient preferences for treatment plans and recognizing AI's potential for treatment monitoring, option identification, and efficiency. Objectification tools in facial exams are valued, with photographic documentation rated as the most vital, alongside provider experience, for patient care improvement. AI's role is seen as pivotal in consultations and follow‐ups, stressing gender‐specific findings and the potential of new facial indices. There's an interest in AI that adapts to diverse patient needs and further research to optimize its use in patient evaluation and personalized treatment planning. (Table 1).

## 4 RESULTS OF CONSENSUS MEETING

The following consents statements received a 100% agreement of the participants.

- AI implemented in aesthetic medicine can help to standardize and improve patient assessment and patient consultation.
- AI implemented in aesthetic medicine can help to prevent overcorrection.
- There is a need for validated objective facial assessments in aesthetic medicine.
- Facial aesthetic assessment should be comprehensive and be based on objective indices (as FAI, FYI or SQI).
- The skin quality assessment should differentiate between female and male skin.
- Patients whose skin is covered with make‐up must be excluded from AI examination at baseline.
- Patients age and gender should be included in the AI assessment.
- Patients' ancestral roots should be included in the AI system.

## 5 RESULTS OF ANCESTRAL ROOTS

A comprehensive analysis of facial features across different ethnic groups—East Asian, Indian, European, Latin and African was presented. It is important to note that our findings should be interpreted in light of the specific patient cases. The findings were as follows: for East Asian roots, the predominant features identified were monolid eyes, square lower face shapes, broader midfaces, a characteristic flat and short nose, and a typically retruded chin. These distinctive morphological traits are emblematic of the East Asian demographic.

In the Indian roots, the consensus highlighted defining traits such as a prominent forehead, brown to black hair, and eye color, as well as significantly larger eyes. Additional features include well‐defined tear troughs and a retruded chin, contributing to the unique facial aesthetic prevalent in individuals of Indian descent.

European faces were noted for having gender‐differentiated eyebrow shapes, with women displaying a peaked brow and men a more horizontal brow line. Common to both genders in this group were features such as a convex forehead, high zygomatic arches, thinner lips, and an inclination towards static wrinkles as a sign of aging.

Participants with Latin American roots exhibited a skin tone spectrum from Fitzpatrick Skin Type II–V and varied eye coloration, ranging from light to very dark brown. Smaller foreheads and a bizygomatic distance equal to or smaller than the bigonial distance were typical. Additionally, the width of the lips was noted to be more than 20% of the total lower facial width, delineating another distinct characteristic of this ethnic group. (Table 2).

**TABLE 2:** Presented characteristics of East Asian, Indian, European, Latin and African ancestral roots.

East Asian face	Indian	European	Latin	African
Monolid Square lowerface Wider midface Flat and short nose Retruded chin	Prominent forehead Brown to black hair and eyes Big eyes Tear trough Retruded chin	Peaked brow in women and horizontal brow in men Average convexity to forehead High zygomatic arch Thinner lips Static wrinkles with age	Skin tone between FST II–V Eye color between light brown, dark brown, and very dark brown Smaller foreheads Bizygomatic distance equal to or smaller than the bigonial distance Width of the lip more than 20% of the lower face width	Darker skin tones with Fitzpatrick Skin Type >III Noses shorter than one‐third of face length, wider than one‐fifth of facial width Lips broader than one‐third of the lower third of the face Forehead wider than one‐third of total facial length Neutral or positive canthal tilt

African facial features commonly include darker skin tones with a Fitzpatrick Skin Type >III. Further, patients with African roots may have noses shorter than one third of the face length, but broader than 1/5 of the facial width.^12^ Such patients also present with lips broader than one third of the lower facial third, as well as a forehead area broader than one third of the facial length.^13^, ^14^ In addition, this group oftentimes shows a neutral or positive canthal tilt, even in elderly patients.^15^

## 6 DISCUSSION

AI's integration into aesthetic medicine is consented as a important for its standardization capabilities, elevating patient assessment and consultation. The unanimity in this regard underscores a recognition of AI's capacity to harmonize evaluations across practitioners, fostering consistency and objectivity that individual expert assessments may lack. This standardization is particularly important in a field where subjectivity has traditionally played a significant role in assessment and decision‐making. Moreover, AI's role in mitigating overcorrection—a common pitfall in aesthetic procedures—has been unanimously recognized. Overcorrection not only compromises the desired natural appearance but can also necessitate corrective interventions, thus elevating risks and costs. AI can meticulously calculate optimal corrections based on vast datasets, reducing the likelihood of human error and the propensity for overly aggressive treatments.

The consensus also robustly endorses the imperative for validated objective facial assessments. The Facial Aesthetic Index (FAI), Facial Youth Index (FYI), and Skin Quality Index (SQI) are highlighted as cornerstones for these assessments.^7^ A comprehensive and objective evaluation is crucial in aesthetic medicine to ensure that treatments align with established indices that quantify the attributes associated with youthful and healthy skin, balanced facial features, and overall aesthetic appeal. By adopting these indices, AI can provide a more nuanced, reproducible, and quantifiable assessment than the subjective evaluations traditionally used.^16^, ^17^

An interesting and critical aspect of the agreement is the emphasis on differentiating skin quality assessments based on gender. Recognizing the biological and physiological differences between male and female skin—such as thickness, texture, and aging patterns—is essential in customizing treatment plans. AI systems that incorporate gender‐specific algorithms can provide more precise and effective recommendations, tailoring to the unique characteristics and needs of each patient.^18^, ^19^

Age has also been unanimously endorsed as critical factors to be included in AI assessments. Age affects the skin's elasticity, collagen production, and overall facial structure, while gender impacts aesthetic preferences and outcomes. The inclusion of these demographics in AI assessments is vital for personalizing treatment plans, ensuring they are appropriate for the patient's life stage and physiological makeup. Perhaps most tellingly, the inclusion of patients' ancestral roots in the AI system has been unanimously agreed upon. This indicates a profound shift towards personalized medicine, accounting for the genetic and phenotypic diversity that influences facial features and skin qualities. An AI system that incorporates ancestral data can better understand and predict individual responses to treatment, align with patients' aesthetic ideals within their roots, and maintain the authenticity of ethnically specific features.

The integration of cultural and ethnic diversity into the AI models is crucial for the equitable assessment of aesthetics across various populations. The consensus findings provide a detailed categorization of facial features across East Asian, Indian, European, Latin and African ancestral lines (Table 2), highlighting the need for individualized assessment and the influence of globalization on beauty standards.

The categorization establishes distinct facial characteristics associated with different ancestral roots. However, the absence of a definitive cutoff for distinguishing between the ancestries suggests that any AI‐driven aesthetic assessment must incorporate a flexible, multi‐dimensional approach to address this complexity. We propose the following considerations for AI algorithm development:

1. *Ethnic and cultural sensitivity in AI training*: AI models should be trained on diverse datasets that are representative of the global population, capturing the full range of human diversity, including those with mixed ancestries. This would require not only a varied dataset but also algorithms capable of learning nuanced differences and preferences within and across cultural contexts.
2. *Case‐by‐case analysis framework*: Given that categorization remains a case‐by‐case decision, AI algorithms should incorporate a decision‐making framework that weighs multiple factors instead of relying on fixed thresholds or rigid classification boundaries.
3. *Globalization's impact on aesthetic standards*: AI models must account for the dynamic nature of beauty standards, which are increasingly influenced by globalization. This entails updating models regularly to reflect current trends while still recognizing traditional and ethnic variations.
4. *Holistic assessment metrics*: Instead of a narrow focus on certain ‘ideal’ features, AI should use comprehensive metrics that evaluate features such as skin quality, facial symmetry, proportionality, and signs of aging in the context of the individual's ethnic background and cultural ideals.
5. *Ethical and bias considerations*: Ethical considerations must be at the forefront of AI aesthetic assessment. Developers should strive to mitigate biases that may arise from a predominance of certain features within training data, which could lead to discrimination or the perpetuation of a narrow definition of beauty.
6. *Interdisciplinary collaboration*: The development of AI algorithms for aesthetic assessment should be an interdisciplinary effort, combining insights from cosmetic surgery, dermatology, anthropology, and psychology, to ensure a holistic and ethically sound approach.

## 7 CONCLUSION

The consensus meeting on AI in aesthetic medicine highlights the promising potential of AI in enhancing patient care and supporting the digital revolution in aesthetic medicine. While practitioners previously relied on conventional methods, they are now recognizing AI's potential to standardize assessments and improve treatment efficiency, while still prioritizing patient preferences. The unanimous agreement on AI's benefits underscores its potential to reduce subjectivity in evaluations and prevent overcorrection in treatments. The need for objective validated facial assessments was highlighted, alongside the importance of personalizing these assessments to account for gender differences and patients' ancestral roots. This personalization is crucial, as it acknowledges the diversity of patient backgrounds and the inadequacies of a one‐size‐fits‐all approach. The proposed model for categorizing ancestral roots has been well‐received, with the understanding that it represents a step towards a more inclusive and detailed framework for patient classification in aesthetic medicine.

## AUTHOR CONRTIBUTIONS

K.F., R.P., and B.R. were involved in the concept and design of the study and drafting of the manuscript. The remaining authors, D.D., J.F., C.C., M.G., S.S., M.K., L.K., A.F., S.C., S.F., K.F., P.P., R.W., and P.H., contributed to the critical revision of the manuscript.

## FUNDING INFORMATION

No funding.

## CONFLICT OF INTEREST STATEMENT

None of the other authors listed have any commercial associations or financial disclosures that might pose or create a conflict of interest with the methods applied or the results presented in this article.

## Data Availability

Data sharing is not applicable to this article as no new data were created or analyzed in this study.

## References

[1] Green JB , Pavicic T , Pooth R , et al. Validated 5–point photonumeric scales for the assessment of hand atrophy. J Cosmet Dermatol. 2022;21(3):933‐939. doi:10.1111/JOCD.14776 35034418

[2] Hayano W , Kerscher M , Day D , et al. Novel, validated, 5‐point Photonumeric scales for assessment of the perioral region. Aesthet Surg J. 2023;43(11):1347‐1356. doi:10.1093/ASJ/SJAD103 37052953

[3] Pavicic T , Pooth R , Prinz V , et al. Validated 5‐point photonumeric scales for the assessment of the periorbital region. J Cosmet Dermatol. 2022;21(1):158‐166. doi:10.1111/JOCD.14643 34865301

[4] Pooth R , Prinz V , Cajkovsky M , et al. Validated 5‐point photonumeric scales for the assessment of the jowls and chin. J Cosmet Dermatol. 2022;21(2):600‐607. doi:10.1111/JOCD.14661 34902199

[5] Carruthers A , Carruthers J , Hardas B , et al. A validated grading scale for marionette lines. Dermatologic Surg. 2008;34 Suppl 2:S167‐S172. doi:10.1111/J.1524-4725.2008.34366.X 19021675

[6] Narins RS , Carruthers J , Flynn TC , et al. Validated assessment scales for the lower face. Dermatologic Surg. 2012;38:333‐342. doi:10.1111/j.1524-4725.2011.02247.x 22316189

[7] Cotofana S , Sattler S , Frank K , et al. Aesthetic medicine‐Quo Vadis? J Cosmet Dermatol. 2022;21:6526‐6527. doi:10.1111/JOCD.15334 36030090

[8] Dorado‐Díaz PI , Sampedro‐Gómez J , Vicente‐Palacios V , Sánchez PL . Applications of artificial intelligence in cardiology. The future is already here. Rev Esp Cardiol (Engl Ed). 2019;72(12):1065‐1075. doi:10.1016/J.REC.2019.05.014 31611150

[9] Sultan AS , Elgharib MA , Tavares T , Jessri M , Basile JR . The use of artificial intelligence, machine learning and deep learning in oncologic histopathology. J Oral Pathol Med. 2020;49(9):849‐856. doi:10.1111/jop.13042 32449232

[10] Girolami I , Pantanowitz L , Marletta S , et al. Artificial intelligence applications for pre‐implantation kidney biopsy pathology practice: a systematic review. J Nephrol. 2022;35(7):1801‐1808. doi:10.1007/s40620-022-01327-8 35441256 PMC9458558

[11] Försch S , Klauschen F , Hufnagl P , Roth W . Artificial intelligence in pathology. Dtsch Arztebl Int. 2021;118(12):199‐204. doi:10.3238/ARZTEBL.M2021.0011 PMC8278129 34024323

[12] Ofodile FA , Bokhari FJ , Ellis C , Matory WE . The Black American nose. Ann Plast Surg. 1993;31(3):209‐219. doi:10.1097/00000637-199309000-00002 8239410

[13] Isiekwe GI , daCosta OO , Isiekwe MC . Lip dimensions of an adult Nigerian population with normal occlusion. J Contemp Dent Pract. 2012;13(2):188‐193. doi:10.5005/JP-JOURNALS-10024-1119 22665746

[14] Codari M , Pucciarelli V , Stangoni F , et al. Facial thirds‐based evaluation of facial asymmetry using stereophotogrammetric devices: application to facial palsy subjects. J Craniomaxillofac Surg. 2017;45(1):76‐81. doi:10.1016/J.JCMS.2016.11.003 27939040

[15] Odunze M , Rosenberg DS , Few JW . Periorbital aging and ethnic considerations: a focus on the lateral canthal complex. Plast Reconstr Surg. 2008;121(3):1002‐1008. doi:10.1097/01.PRS.0000299381.40232.79 18317149

[16] Elder A , Ring C , Heitmiller K , Gabriel Z , Saedi N . The role of artificial intelligence in cosmetic dermatology‐current, upcoming, and future trends. J Cosmet Dermatol. 2021;20(1):48‐52. doi:10.1111/JOCD.13797 33151612

[17] Buzzaccarini G , Degliuomini RS , Borin M . The artificial intelligence application in aesthetic medicine: how ChatGPT can revolutionize the aesthetic world. Aesth Plast Surg. 2023;47(5):2211‐2212. doi:10.1007/S00266-023-03416-W 37256297

[18] Barone M , De Bernardis R , Persichetti P . Artificial intelligence in plastic surgery: analysis of applications, perspectives, and psychological impact. Aesth Plast Surg. 2024;94:38‐39. doi:10.1007/S00266-024-03988-1 38472350

[19] Atiyeh B , Emsieh S , Hakim C , Chalhoub R . A narrative review of artificial intelligence (AI) for objective assessment of aesthetic endpoints in plastic surgery. Aesth Plast Surg. 2023;47(6):2862‐2873. doi:10.1007/S00266-023-03328-9 37000298

